# Higher radiation dose on immune cells is associated with radiation-induced lymphopenia and worse prognosis in patients with locally advanced esophageal squamous cell carcinoma

**DOI:** 10.3389/fimmu.2023.1066255

**Published:** 2023-05-08

**Authors:** Jianjian Qiu, Hancui Lin, Dongmei Ke, Yilin Yu, Jiaying Xu, Hejin Qiu, Qunhao Zheng, Hui Li, Hongying Zheng, Lingyun Liu, Zhiping Wang, Qiwei Yao, Jiancheng Li

**Affiliations:** Clinical Oncology School of Fujian Medical University, Fujian Cancer Hospital, Fuzhou, China

**Keywords:** effective dose to immune cells (EDIC), esophageal carcinoma, radiation-induced lymphopenia (RIL), prognosis, radiotherapy

## Abstract

**Background:**

To explore the effective dose to immune cells (EDIC) for better prognosis while avoiding radiation-induced lymphopenia (RIL) in patients with locally advanced esophageal squamous cell carcinoma (ESCC).

**Materials and methods:**

Overall, 381 patients with locally advanced ESCC receiving definitive radiotherapy with or without chemotherapy (dRT ± CT) between 2014 and 2020 were included in this study. The EDIC model was calculated by radiation fraction number and mean doses to the heart, lung, and integral body. The correlation between EDIC and clinical outcomes was analyzed using Cox proportional hazards regression, and risk factors for RIL were determined by logistic regression analysis.

**Results:**

The median EDIC was 4.38 Gy. Multivariate analysis revealed that low-EDIC significantly improved the OS of patients when compared with high-EDIC (HR = 1.614, P = 0.003) and PFS (HR = 1.401, P = 0.022). Moreover, high-EDIC was associated with a higher incidence of grade 4 RIL (OR = 2.053, P = 0.007) than low-EDIC. In addition, we identified body mass index (BMI), tumor thickness, and nodal stage as independent prognostic factors of OS and PFS, while BMI (OR = 0.576, P = 0.046) and weight loss (OR = 2.214, P = 0.005) as independent risk factors of grade 4 RIL. In subgroup analyses, the good group had better clinical outcomes than the remaining two groups (P< 0.001).

**Conclusion:**

This study demonstrated that EDIC significantly correlates with poor clinical outcomes and severe RIL. Optimizing treatment plans to decrease the radiation doses to immune cells is critical for improving the outcomes.

## Introduction

Esophageal cancer (EC) is among the most common malignancies in China and worldwide, with 3 million deaths annually (1–3). However, most patients are diagnosed at an advanced stage due to a lack of effective screening methods for early-stage EC (4, 5). Radiotherapy is an essential treatment strategy for patients with locally advanced EC and there has been great progress in it. Nonetheless, the prognosis of locally advanced EC remains unsatisfactory (6), with a 5-year overall survival (OS) rate of 15%–25% worldwide (7). Fortunately, immunotherapy has shown enormous promise in clinical trials, and is used in clinical practice with a dramatically improved survival rate of patients with lung cancer and malignant melanoma (8–13). In addition, the development of monoclonal antibodies, anti-programmed death-1 (PD-1) and anti-programmed death-ligand 1 (PD-L1), has produced a significant therapeutic response in EC (14). Hence, radiotherapy can be combined with immunotherapy as a novel treatment strategy for patients with EC.

The immune system is crucial for promoting tumorigenesis. However, thoracic radiotherapy alters the immune system function and thus affects tumor control. On the one hand, it stimulates the immune system by releasing specific antigens and cascade reactions of atypical cytokine signals, thereby limiting tumor growth and metastasis (15). The abscopal effect, which is tumor shrinkage outside the radiation fields, has confirmed this theory observed in animal experiments and clinical practice (16–18). On the other hand, since lymphocytes are sensitive to radiation (19, 20), radiotherapy can suppress the immune function by killing them, thereby reducing the therapeutic effect. Moreover, previous studies revealed that radiation-induced lymphopenia (RIL) is associated with poor prognosis (19, 21). Additionally, increasing the radiation dose to the tumor increases the radiation dose to immune cells. To this end, a clinical trial considered the immune system as a risky organ to calculate the effective dose to immune cells (EDIC) and found that the radiation dose of the immune system was associated with OS and local tumor control (22). Consistently, another study of non-small cell lung cancer also proved the relationship between EDIC and the prognosis of patients (23). However, there are only a few studies on this aspect in esophageal squamous cell carcinoma (ESCC). Therefore, we aimed to apply a model to calculate the EDIC, and explore the relationship of EDIC with clinical outcomes and RIL in patients with locally advanced ESCC.

## Materials and methods

### Study population

We included 381 patients with locally advanced ESCC who underwent definitive radiotherapy with/without chemotherapy at Fujian Medical University Cancer Hospital between 2014 and 2020 in this study. This study complied with the Declaration of Helsinki and was approved by the Institutional Ethics Committee. The inclusion criteria were as follows (1): Cytologically or pathologically confirmed ESCC (2), age > 18 years (3), treatment with definitive radiotherapy (≥ 50 Gy and ≥ 25 fractions) (4), no distinct metastasis or other malignancies (5), no surgery, and (6) available clinicopathologic and follow-up data. All patients were staged according to the 8^th^ version of AJCC.

### Treatments and follow-up

Several radiotherapy oncologists comprehensively evaluated the auxiliary examinations of patients before the initiation of the treatment, under the guidance of clinical practice guidelines. All patients underwent individualized thoracic radiotherapy with either intensity-modulated radiation therapy (IMRT) or 3-dimensional conformal radiation therapy (3D-CRT). The radiation dose prescriptions were 50–70 Gy in 25–34 fractions, five days per week. The gross tumor volume (GTV) included primary esophageal tumor and involved lymph nodes. Due to micrometastasis, the clinical target volume (CTV) included GTV of ≥ 3 cm in the upper and lower margin, and 0.5 cm in the lateral margin. Based on CTV, the planning target volume (PTV) expanded by a 0.5–1 cm margin. According to the 2019 esophageal carcinoma Guidelines of the National Comprehensive Cancer Network, the plans of all patients must meet the dose-volume limitations for organs at risk (OARS). All patients received 0–7 cycles of sequential or concurrent chemotherapy. Chemotherapy regimens included docetaxel, paclitaxel + nedaplatin, cisplatin, lobaplatin or carboplatin, and 5-fluorouracil + cisplatin. The patients were followed up every three months in the first year, every six months after the second year, and then annually. Follow-up was to monitor the patient’s survival status and disease changes, and the median follow-up time was 21 months.

### Data collection

We extracted clinical features, including gender, age, weight change, body mass index (BMI), chemotherapy regimens, chemotherapy cycles, tumor location, tumor length, tumor thickness, TNM stage, and complete blood count (CBC), from electronic medical records. In this study, patients with a weight loss of 1 kg or more from their usual weight at diagnosis were defined as having lost weight (within 1 month). The Pinnacle system was used to obtain the dose-volume histogram (DVH) data of patients. Mean lung dose (MLD), mean heart dose (MHD), and mean body dose (MBD) were used to calculate the EDIC of the patient. In this study, the lymphocyte count was collected before and during radiotherapy (weekly). The minimum lymphocyte count during thoracic radiotherapy was defined as the lymphocyte nadir. The Common Terminology Criteria for Adverse Events (CTCAE) version 4.0 graded RIL.

### Calculation of EDIC

EDIC estimates the dose to immune cells by using the radiation dose to circulation blood as a surrogate (22, 23). The circulation blood pool that is irradiated in radiotherapy includes the heart, lungs, and the large and small vessels/capillaries in the remaining organs. Additionally, the components were estimated from anatomy/physiology textbooks to estimate the percentage of cardiac output and blood volume for each component. The heart and lungs account for about 8% and 12% of the cardiac output, respectively. In addition, the blood volume in great vessels and small vessels account for 45% and 35% of cardiac output, respectively. The dose effectiveness factor for small vessels was 0.85. The final model was developed based on the following equation for patients undergoing ≥ 25 fractions of thoracic radiation:


$$\text{EDIC }=\;0.12*\text{MLD }+\;0.08*\text{MHD }+\;\left[0.45\;+\;0.35*0.85*{\left(\text{n}/45\right)}^{1/2}*\text{MBD}\right]$$

### Statistical analysis

The primary endpoint was OS, which was defined from the date of pathological diagnosis to death due to any cause or last follow-up. The secondary endpoint was PFS, calculated from the date of pathological diagnosis to disease progression, death, or last follow-up. The Kaplan–Meier (KM) method was used to estimate the survival curves and univariate cox analysis to identify the crucial clinical factors that affect survival outcomes. The covariates with a P value< 0.05 in univariate analysis were incorporated into the multivariate analysis, which identified the independent prognostic factors. We evaluated the correlations among independent prognostic factors using Spearman correlation analysis. The logistic regression analysis was used to identify the potential risk factors with grade 4 RIL. The receiver operating characteristics (ROC) curve computed the optimal cut-off values of BMI, tumor length, tumor thickness, and EDIC. All statistical analyses were two-sided, and P value of < 0.05 was considered statistically significant. All statistical analyses were performed using SPSS software (version 25.0).

## Results

### Patient characteristics

In all, we included 381 patients in the final analysis and the clinical characteristics are summarized in **Table 1**. The median age of patients was 67 and 69.6% were males. Approximately 49.6% of patients experienced weight loss. Chemotherapy accounted for 75.1% and 56.4% of patients received concurrent chemoradiotherapy. Most common primary tumors were located in the upper (34.4%) and lower (48.3%) thorax. About 23.1% of the patients were at stage II, 29.4% were at stage III, and 47.5% at were stage IVA. The rates of grades 1, 2, 3, and 4 RIL were 1.3%, 15.0%, 62.2%, and 21.5%. The cut-off values for BMI, tumor length, tumor thickness, and EDIC were 19.03, 5.9 cm, 1.7 cm, and 4.78 Gy.

**Table 1:** Patient clinical characteristics.

Characteristics		No.of patients (n = 381)
Age (years)		
	< 67	180 (47.2%)
	≥ 67	201 (52.8%)
Gender		
	Male	265 (69.6%)
	Female	116 (30.4%)
Weight loss		
	No	192 (50.4%)
	Yes	189 (49.6%)
BMI		
	< 19.03	100 (26.2%)
	≥ 19.03	281 (73.8%)
Radiotherapy		
	IMRT	299 (78.5%)
	3D-CRT	82 (21.5%)
Chemotherapy		
	concurrent chemotherapy	215 (56.4%)
	sequential chemotherapy	71(18.6%)
	without chemotherapy	95 (24.9%)
Chemotherapy regimen		
	paclitaxel + platinum	198(52.0%)
	5-fluorouraci + platinum	50(13.1%)
	docetaxel + platinum	38(10.0%)
	no	95 (24.9%)
Tumor location		
	Cervical	37 (9.7%)
	Upper thoracic	131 (34.4%)
	Middle thoracic	29 (7.6%)
	Lower thoracic	184 (48.3%)
Tumor length (cm)		
	< 5.9	209 (54.9%)
	≥ 5.9	172 (45.1%)
Tumor thickness (cm)		
	< 1.7	276 (72.4%)
	≥ 1.7	105 (27.6%)
T stage		
	T2	26 (6.8%)
	T3	188 (49.3%)
	T4	167 (43.8%)
N stage		
	N0	111 (29.1%)
	N1	165 (43.3%)
	N2	83 (21.8%)
	N3	22 (5.8%)
TNM stage		
	Stage II	88 (23.1%)
	Stage III	112 (29.4%)
	Stage IVA	181 (47.5%)
Radiation-induced lymphopenia		
	Grade 1	5 (1.3%)
	Grade 2	57 (15.0%)
	Grade 3	237 (62.2%)
	Grade 4	82 (21.5%)
EDIC		
	< 4.78	217 (57.0%)
	≥ 4.78	164 (43.0%)

BMI, body mass index; IMRT, intensity-modulated radiation therapy; 3D-CRT, 3-dimensional conformal radiation therapy; T, tumor; N, node; TNM, tumor-node-metastasis; EDIC, effective dose to the immune cell; RIL, radiation-induced lymphopenia.

### Prognostic factors of OS and PFS

**Figure 1:**
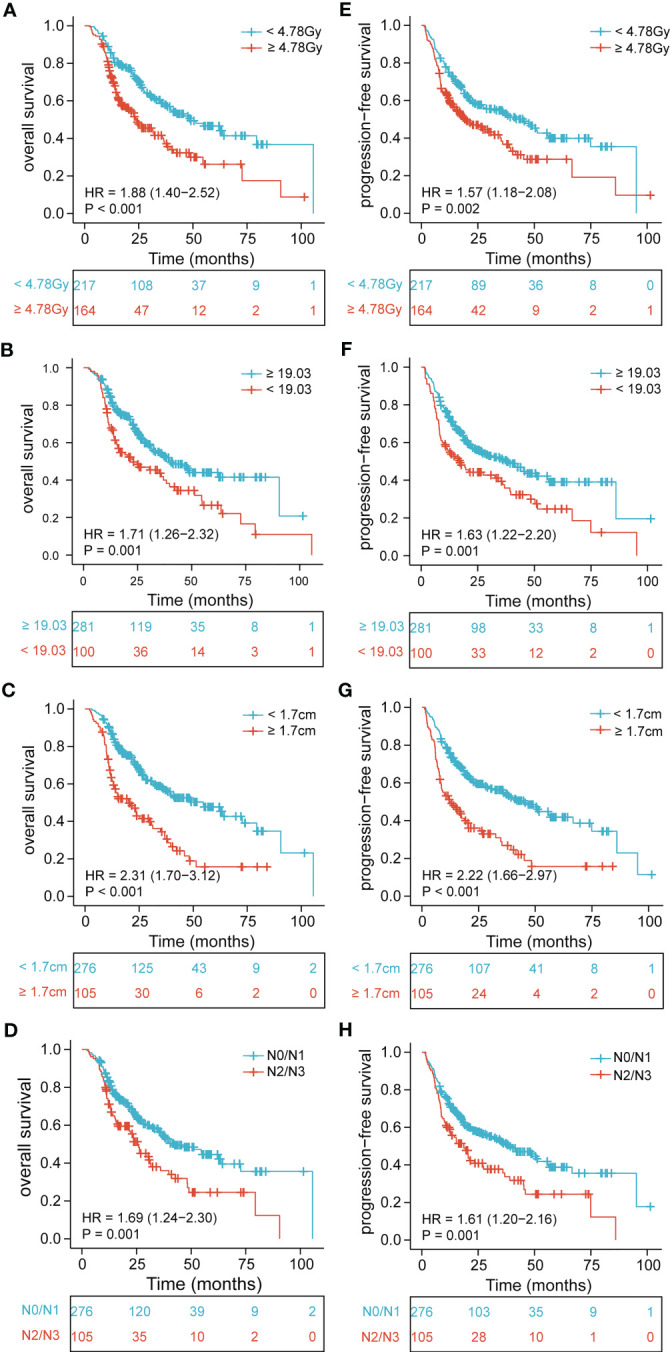
Kaplan-Meier curves of EDIC, BMI, tumor thickness, and N stage for all patients showing **(A–D)** overall survival (p< 0.001, p = 0.001, p< 0.001, p = 0.001, respectively); **(E–H)** progression-free survival (p = 0.002, p = 0.001, p< 0.001, p = 0.001, respectively). EDIC, effective dose to the immune cell; BMI, body mass index; HR, hazard ratio; N, node.

The median OS and PFS were 21 months (range, 2.1–105.6 months) and 17.2 months (range, 1.2–101.4 months), respectively. Using univariate analysis, we identified BMI (P = 0.001), tumor location (P = 0.025), tumor length (P< 0.001), tumor thickness (P< 0.001), N stage (P = 0.001), TNM stage (P = 0.004), and EDIC (P< 0.001) as significant prognostic factors of a worse OS (**Table 2**). Of these, the multivariate analysis identified BMI (HR = 0.619, 95%CI, 0.452-0.848, p = 0.003), tumor thickness (HR = 1.859, 95% CI: 1.313–2.630, P< 0.001), N stage (HR = 1.534, 95% CI: 1.102–2.134, P = 0.011), and EDIC (HR = 1.614, 95% CI, 1.176–2.215, P = 0.003) as independent risk factors of OS. Additionally, univariate analysis recognized BMI (P = 0.001), tumor length (P< 0.001), tumor thickness (P< 0.001), N stage (P = 0.001), TNM stage (P< 0.001), and EDIC (p = 0.002) as significant prognostic factors of a worse PFS (**Table 3**). On multivariate analysis, BMI (HR = 0.667, 95% CI: 0.494–0.900, P = 0.008), tumor thickness (HR = 1.797, 95% CI: 1.282–2.517, P = 0.001), N stage (HR = 1.396, 95% CI: 1.021–1.910, P = 0.037), and EDIC (HR = 1.401, 95% CI: 1.050–1.869, P = 0.022) were identified as independent risk factors of PFS. After adjusting for other risk factors, EDIC was identified as a significant prognostic factor for both, OS and PFS. Spearman’s analysis results showed that there is no correlation or weak correlation between the prognostic factors (correlation coefficient: 0.150 - 0.207). As shown in **Figure 1**, there were noteworthy differences in the OS and PFS in EDIC, BMI, tumor thickness, and N stage.

**Table 2:** Univariate and multivariate cox regression analysis of patient clinical characteristics with overall survival.

Characteristics	Univariate analysis			Multivariate analysis		
	HR	95% CI	P value	HR	95% CI	P value
Age (years)						
≥ 67 vs< 67	1.343	0.998-1.808	0.052			
Gender						
Female vs Male	1.108	0.805-1.618	0.220			
Weight loss						
Yes vs No	1.247	0.930-1.673	0.141			
BMI						
≥ 19.03 vs< 19.03	0.586	0.432-0.797	0.001	0.619	0.452-0.848	0.003
Radiotherapy						
IMRT vs 3D-CRT	1.187	0.818-1.723	0.368			
Chemotherapy						
Concurrent vs sequential vs without	1.092	0.917-1.300	0.325			
Tumor location						
Cervical/Upper vs Middle/Lower	1.411	1.044-1.908	0.025	1.162	0.845-1.598	0.357
Tumor length (cm)						
≥ 5.9 vs< 5.9	1.921	1.428-2.585	< 0.001	1.296	0.920-1.825	0.138
Tumor thickness (cm)						
≥ 1.7 vs< 1.7	2.306	1.704-3.121	< 0.001	1.859	1.313-2.630	< 0.001
T stage						
T4 vs T2/T3	1.112	0.830-1.491	0.477			
N stage						
N2/N3 vs N0/N1	1.691	1.244-2.298	0.001	1.534	1.102-2.134	0.011
TNM stage						
Stage III/Stage IVA vs Stage II	1.818	1.207-2.741	0.004	1.173	0.752-1.830	0.482
EDIC						
≥ 4.78 vs< 4.78	1.879	1.398-2.524	< 0.001	1.614	1.176-2.215	0.003
RIL						
Grade 4 vs Grade ≤3	1.141	0.805-1.618	0.458			

HR, hazard ratio; CI, confidence interval; BMI, body mass index; IMRT, intensity-modulated radiation therapy; 3D-CRT, 3-dimensional conformal radiation therapy; T, tumor; N, node; TNM, tumor-node-metastasis; EDIC, effective dose to the immune cell; RIL, radiation-induced lymphopenia.

**Table 3:** Univariate and multivariate cox regression analysis of patient clinical characteristics with progression free-survival.

Characteristics	Univariate analysis			Multivariate analysis		
	HR	95% CI	P value	HR	95% CI	P value
Age (years)						
≥ 67 vs< 67	1.189	0.897-1.574	0.229			
Gender						
Female vs Male	1.091	0.935-1.274	0.268			
Weight loss						
Yes vs No	1.297	0.980-1.717	0.069			
BMI						
≥ 19.03 vs< 19.03	0.612	0.456-0.822	0.001	0.667	0.494-0.900	0.008
Radiotherapy						
IMRT vs 3D-CRT	1.023	0.714-1.466	0.902			
Chemotherapy						
Concurrent vs sequential vs without	1.034	0.875-1.223	0.692			
Tumor location						
Cervical/Upper vs Middle/Lower	1.261	0.949-1.676	0.110			
Tumor length (cm)						
≥ 5.9 vs< 5.9	1.841	1.389-2.441	< 0.001	1.238	0.893-1.718	0.201
Tumor thickness (cm)						
≥ 1.7 vs< 1.7	2.219	1.659-2.969	< 0.001	1.797	1.282-2.517	0.001
T stage						
T4 vs T2/T3	1.231	0.932-1.626	0.143			
N stage						
N2/N3 vs N0/N1	1.613	1.202-2.164	0.001	1.396	1.021-1.910	0.037
TNM stage						
Stage III/Stage IVA vs Stage II	2.038	1.365-3.042	< 0.001	1.421	0.923-2.186	0.110
EDIC						
≥ 4.78 vs< 4.78	1.566	1.182-2.075	0.002	1.401	1.050-1.869	0.022
RIL						
Grade 4 vs Grade ≤3	1.053	0.751-1.477	0.766			

HR, hazard ratio; CI, confidence interval; BMI, body mass index; IMRT, intensity-modulated radiation therapy; 3D-CRT, 3-dimensional conformal radiation therapy; T, tumor; N, node; TNM, tumor-node-metastasis; EDIC, effective dose to the immune cell; RIL, radiation-induced lymphopenia.

**Figure 2:**
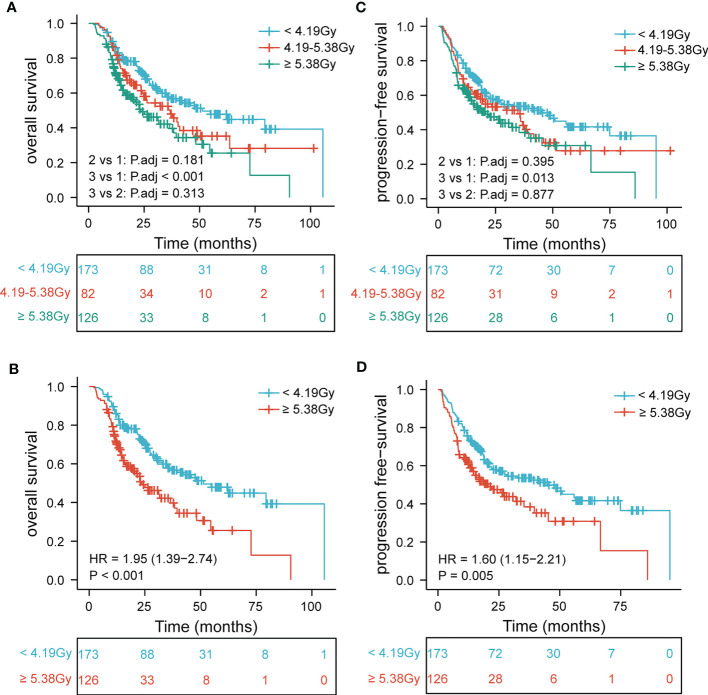
Patients are stratified by EDIC cut-off values. Kaplan-Meier curves for **(A)** overall survival by three categories; **(B)** overall survival first versus third; **(C)** progression-free survival by three categories; **(D)** progression-free survival first versus third. EDIC, effective dose to the immune cell; R, hazard ratio.

**Figure 3:**
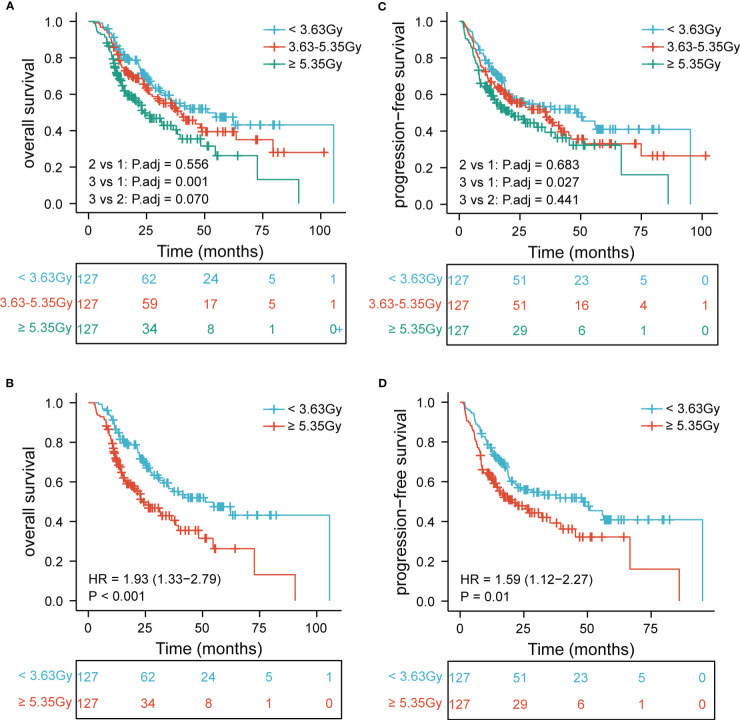
Patients are stratified by the equal study population. Kaplan-Meier curves for **(A)** overall survival by three categories; **(B)** overall survival first versus third; **(C)** progression-free survival by three categories; **(D)** progression-free survival first versus third. EDIC, effective dose to the immune cell; R, hazard ratio.

Further, to determine the association of EDIC with clinical outcomes, we divided EDIC into three categories according to cut-off values (< 4.19 Gy, 4.19–5.38 Gy, and ≥ 5.38 Gy) and equal study population (< 3.63 Gy, 3.63–5.35 Gy, and ≥ 5.35 Gy). The OS and PFS rates for EDIC divided into three groups based on the cut-off values are shown in **Figure 2** (P< 0.001 and P = 0.0014). Patients with EDIC ≥ 5.38 Gy had significantly worse OS and PFS than those with EDIC< 4.19 Gy (P< 0.001 and P = 0.005). Comparisons between other groups were not statistically significant. The median OS for patients with EDIC ≥ 5.38 Gy and< 4.19 Gy were 23.6 and 51.3 months, respectively. The median PFS for patients with EDIC ≥ 5.38 Gy and< 4.19 Gy were 20.6 and 45.1 months, respectively. Furthermore, EDIC was divided into three groups according to the equal study population. The OS and PFS curves of the two EDICs are shown in **Figure 3** (P = 0.001 and P = 0.0029). Patients with EDIC ≥ 5.35 Gy had significantly worse OS and PFS than those with EDIC< 3.63 Gy (P = 0.001 and P = 0.029). Both approaches showed a strong correlation of EDIC scores with OS and PFS.

### Survival is stratified by EDIC, BMI, tumor thickness, and N stage

**Figure 4:**
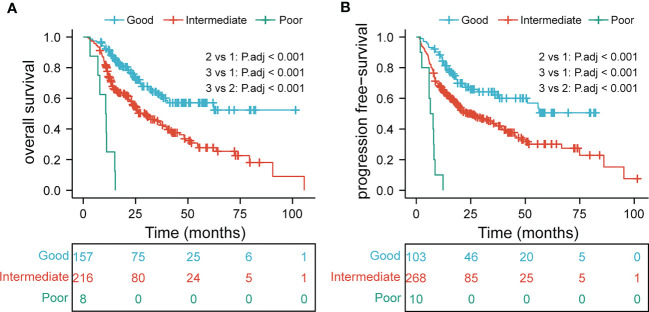
overall survival **(A)** and progression-free survival **(B)** in subgroup analysis based on multivariate analysis results.

The EDIC, BMI, tumor thickness, and N stage were crucial prognostic factors for survival. Patients with EDIC ≥ 4.78 Gy, BMI< 19.03, tumor thickness ≥ 1.7 cm, and N2/N3 were considered to have one score. We observed that the lower the score, the worse the prognosis. Then, we divided the patients into three groups based on the independent prognostic factors: the poor group (0–1 scores), the intermediate group (2 scores), and the good group (≥ 3 scores). KM curves showed prominent differences in the OS (P<0.001) and PFS (P<0.001) among the three groups (**Figure 4**).

### Risk factors of RIL

The lymphocyte count declined remarkably during thoracic radiotherapy, and the median lymphocyte nadir was 0.4*10^9^/L. Univariate logistic analysis showed that lower EDIC was associated with a higher lymphocyte count (P = 0.004). The multivariate logistic regression analysis revealed that EDIC was significantly correlated with RIL (OR, 2.053, 95% CI: 1.221–3.451, P = 0.007) after adjusting for other confounding variables. In addition, the BMI (P = 0.046) and weight loss (P = 0.005) were independent risk factors of RIL (**Table 4**).

**Table 4:** Univariate and multivariate logistic regression analysis for radiation-induced lymphopenia.

Characteristics	Univariate analysis			Multivariate analysis		
	OR	95% CI	P value	OR	95% CI	P value
Age (years)						
≥ 67 vs < 67	0.984	0.603-1.605	0.948			
Gender						
Female vs Male	1.074	0.629-1.835	0.794			
Weight loss						
Yes vs No	2.050	1.239-3.392	0.005	2.214	1.258-3.586	0.005
BMI						
≥ 19.03 vs < 19.03	0.494	0.293-0.831	0.008	0.576	0.335-0.989	0.046
Radiotherapy						
IMRT vs 3D-CRT	0.942	0.516-1.716	0.844			
Chemotherapy						
Concurrent vs sequential vs without	0.872	0.648-1.172	0.363			
Tumor location						
Cervical/Upper vs Middle/Lower	1.486	0.898-2.458	0.123			
Tumor length (cm)						
≥ 5.9 vs < 5.9	1.992	1.213-3.272	0.006	1.646	0.982-2.758	0.059
Tumor thickness (cm)						
≥ 1.7 vs < 1.7	1.293	0.760-2.200	0.343			
T stage						
T4 vs T2/T3	0.829	0.505-1.363	0.460			
N stage						
N2/N3 vs N0/N1	1.495	0.884-2.527	0.133			
TNM stage						
Stage III/Stage IVA vs Stage II	1.308	0.713-2.401	0.386			
EDIC						
≥ 4.78 vs < 4.78	2.089	1.273-3.428	0.004	2.053	1.221-3.451	0.007

OR, odds ratio; CI, confidence interval; BMI, body mass index; IMRT, intensity-modulated radiation therapy; 3D-CRT, 3-dimensional conformal radiation therapy; T, tumor; N, node; TNM, tumor-node-metastasis; EDIC, effective dose to the immune cell.

## Discussion

This study included 381 patients with locally advanced ESCC and revealed that EDIC is correlated with RIL during thoracic radiotherapy, and is an important prognostic factor for both, OS and PFS. These findings implicate that undue radiation doses to immune cells, especially lymphocytes, lead to severe lymphopenia and poor clinical outcomes.

Previous studies reported that high heart and lung radiation doses are significantly associated with decreased OS (24, 25). Someone argued that heart and lung toxicity leads to poor survival. However, according to the RTOG 0617 trial, the high-dose group had lower heart and lung toxicity than the low-dose group (22). Additionally, in multivariate cox analysis, MHD was significantly associated with local recurrence-free survival (LRFS), while MLD was important for PFS. This suggests that MHD and MLD were correlated with survival because of disease control or progression and not toxicity (22). Instead, MHD and MLD may be a surrogate for radiation dose to circulating lymphocytes in blood and are vital for tumor development. Therefore, the EDIC model was developed to predict the dose to circulating lymphocytes from the mean heart, lung, and body doses.

Circulating lymphocytes are among the most radiosensitive cells with a D50 (dose required for 50% pre-treatment lymphocyte cell death) of approximately 2 Gy. RIL is a common phenomenon observed during radiotherapy. The significance of EDIC for RIL in this study is consistent with previous findings (20) and can be explained by the principles of radiobiology. Moreover, several studies demonstrated that dosimetric factors, such as heart V50 and lung V5 in lung cancer and mean body dose in EC, are essential risk factors (19, 26, 27).

Radiotherapy causes immunosuppression by killing circulating lymphocytes in many solid tumor treatments. Thus, it could theoretically reduce the treatment efficacy and affect the prognosis, and is considered a negative prognostic factor in malignant solid tumors (19, 28). Nonetheless, the decrease in lymphocyte count after irradiation is not always associated with poor post-treatment survival outcomes. For instance, in a study of 395 EC patients, the 5-year OS difference between grade 4 and non-grade 4 lymphopenia was not statistically significant (29). Similarly, 83% of the patients with oropharyngeal cancer receiving definitive CRT at the MD Anderson Cancer Center had ≥ grade 3 and 25% had grade 4 lymphopenia, which did not affect the survival or local control outcomes (30). Similarly, Holub et al. did not find an association between post-treatment lymphopenia and survival outcomes (31). Likewise, we observed no difference in OS and PFS between grade 4 RIL compared with grades 1–3. The median survival of patients with grade 1–3 RIL was 6 months longer than those with grade 4 (36.8 vs. 30.4 months); however, the difference was not statistically significant. Therefore, the relationship between RIL and prognosis is unclear. Several reasons may account for the negative results in this study. Firstly, in addition to direct damage to lymphocytes by RT, lymphocytes infiltrating from peripheral blood after RT stimulation might also contribute to circulating lymphopenia. Second, the radiosensitivity of lymphocytes also represents the radiosensitivity of cancer cells, which predicts better survival (32). Third, there may be a bias in the lymphocyte nadir because more than half of the patients in this research received chemotherapy, which often has considerable hematological toxicity, especially for patients receiving concurrent chemoradiotherapy. Finally, since lymphocyte changes dynamically during radiotherapy, it is difficult to evaluate the immune status of patients using only the lymphocyte nadir, which results in negative results.

Our study confirmed the association between EDIC and RIL and revealed the impact of EDIC on the survival of patients with locally advanced ESCC. Consistently, previous studies demonstrated that high EDIC was an important risk factor for OS, PFS, and disease-free survival in lung cancer (23, 33). This may be due to the radiation-induced damage to immune cells, which are vital for limiting metastatic growth and maintaining the spreading cancer cells in an inert state (34–36). Tumor progression is the leading cause of death in patients with cancer. Although EDIC is an objective variable influenced by radiotherapy planning, its potential determinants, such as tumor size and N stage, were not considered, which may be related to clinical outcomes. The number of positive lymph nodes and tumor size are negatively associated with the survival of patients with EC (37). In addition, tumor size and N stage can affect the radiation area and dose during the development of radiotherapy schedules. Large GTV was a risk factor for worse OS and PFS in previous studies (38, 39). However, after adjusting for GTV size effects, a second study of RTOG0617 data revealed that EDIC was still substantially linked with OS and LPFS (22). Another study suggested that PTV did not correlate with OS or LPFS (23). Interestingly, spearman’s analysis results showed that there is no correlation or weak correlation between EDIC and tumor thickness or N stage in this study. In all, the survival significance of EDIC may provide new insights into treatment schedule optimization in our daily practice.

The EDIC scoring is a powerful tool to assist clinicians in identifying high-risk patients for early intervention. It is a combined influence of beam-on time, radiation dose, and immune cell fractions. Hence, several approaches related to these factors can potentially decrease EDIC. Advanced radiotherapy techniques, such as high-dose, hypofractionated SBRT, and high-dose-rate FLASH RT, reduce radiation delivery time thereby decreasing the circulating blood exposure (40). Moreover, proton beam therapy has better dose distribution and significantly lowers the dose in surrounding normal tissues than IMRT. Near the heart and lungs can drop the dose sharply (41). Of course, there are other advanced radiotherapy technologies, such as image-guided adaptive therapy and heavy ions therapy. From the perspective of clinicians, it is important to optimize planning by adjusting beam energy and direction and the number of beams before therapy. In addition, to accommodate anatomical changes and tumor regression, we may need to optimize the treatment plan again.

This study has certain limitations. Firstly, since it was a retrospective study, there was inevitable selection bias and did not consider all confounding factors, such as chemotherapy regimen, radiotherapy dose, and target volume size (such as GTV or PTV). Secondly, the EDIC equation only considered the estimate of circulating or resident immune cell pools in large organs within the radiation field, including the heart, lungs, liver, and kidneys. It did not incorporate the contributions of lymphatic vessels, lymph nodes, thymus, spleen, and bone marrow. Hence, it may not fully represent of the influence of radiation on the immune cells. Although the contribution of bone marrow to acute lymphopenia is small, it plays a role in lymphocyte recovery after treatment. The adult thymus is degenerated, hence its contribution to the associated lymphocyte pool is small. Additionally, due to anatomical location, the radiation dose to the spleen in thoracic radiotherapy is limited and has little effect on lymphopenia. Therefore, we need to refine the EDIC model by including lymphoid structures and other related organs. Lastly, this is a single-center, small-sample study that needs to be validated by a prospective multicenter study with a larger sample size.

## Conclusion

This study identified a correlation of EDIC with poor clinical outcomes and severe RIL, which indicates that high doses to the immune system were related to tumor progression and death. Hence, it is important to optimize treatment plans to decrease the radiation doses to immune cells for improving the clinical outcomes.

## Data availability statement

The raw data supporting the conclusions of this article will be made available by the authors, without undue reservation.

## Ethics statement

The studies involving human participants were reviewed and approved by the ethics committee of Fujian Medical University Cancer Hospital. The patients/participants provided their written informed consent to participate in this study.

## Author contributions

QY, ZW and JQ designed this study. JQ, DK, YY, HQ, and JX contributed to data collection. HCL, HL, and QZ analyzed the data. JL and QY supervised the study. JQ, LL, HZ, and ZW wrote the manuscript. All authors contributed to the article and approved the submitted version.
